# Complement System in Dermatological Diseases – Fire Under the Skin

**DOI:** 10.3389/fmed.2015.00003

**Published:** 2015-01-29

**Authors:** Jaana Panelius, Seppo Meri

**Affiliations:** ^1^Department of Bacteriology and Immunology, Haartman Institute, University of Helsinki, Helsinki, Finland; ^2^Department of Dermatology and Allergology, Skin and Allergy Hospital, Helsinki University Central Hospital, Helsinki, Finland; ^3^Huslab, Helsinki University Central Hospital, Helsinki, Finland; ^4^Research Programs Unit, Immunobiology, University of Helsinki, Helsinki, Finland

**Keywords:** hereditary and acquired angioedema, systemic lupus erythematosus, vasculitic syndromes, pemphigus, pemphigoid, partial lipodystrophy, complement deficiency, complement evasion

## Abstract

The complement system plays a key role in several dermatological diseases. Overactivation, deficiency, or abnormality of the control proteins are often related to a skin disease. Autoimmune mechanisms with autoantibodies and a cytotoxic effect of the complement membrane attack complex on epidermal or vascular cells can cause direct tissue damage and inflammation, e.g., in systemic lupus erythematosus (SLE), phospholipid antibody syndrome, and bullous skin diseases like pemphigoid. By evading complement attack, some microbes like *Borrelia* spirochetes and staphylococci can persist in the skin and cause prolonged symptoms. In this review, we present the most important skin diseases connected to abnormalities in the function of the complement system. Drugs having an effect on the complement system are also briefly described. On one hand, drugs with free hydroxyl on amino groups (e.g., hydralazine, procainamide) could interact with C4A, C4B, or C3 and cause an SLE-like disease. On the other hand, progress in studies on complement has led to novel anti-complement drugs (recombinant C1-inhibitor and anti-C5 antibody, eculizumab) that could alleviate symptoms in diseases associated with excessive complement activation. The main theme of the manuscript is to show how relevant the complement system is as an immune effector system in contributing to tissue injury and inflammation in a broad range of skin disorders.

## Introduction

**Figure 1:**
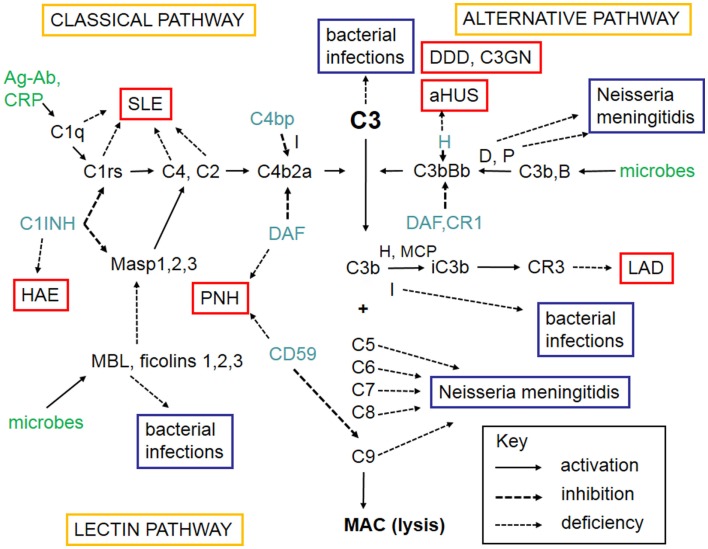
**A schematic figure of the complement system**. The figure shows the three complement activation pathways (yellow boxes), the components needed for activation (in black, like C3), the targets that activate complement (green), the different complement regulators (blue), and consequences of deficiencies of individual complement components leading either to infections (lilac boxes) or to complement deficiency syndromes (in red boxes). Explanations of the different arrow types are shown in the key box (bottom right). The classical pathway (top left) can be activated by C1q that binds to immune complexes or C-reactive protein. The lectin pathway (bottom left) becomes activated by mannan-binding lectin (MBL) or ficolins that bind to carbohydrates or acetylated moieties. The alternative pathway (top right) becomes activated spontaneously upon interaction with a foreign surface (e.g., microbes) that lacks complement inhibitors. After binding of C1q, MBL, or ficolins to their targets, the serine esterases attached to them (C1r, C1s, MASPs) become activated and cleave the subsequent components C4 and C2. C4b and C2a together generate the classical pathway C3/C5 convertase. Activation of C3 is central to complement activation. It can become activated by the alternative pathway C3/C5 convertase C3bBb composed of C3b and the activated factor B. Factor D activates factor B and properdin (P) stabilizes the C3bBb enzyme. Because of involvement of C3b in C3 cleavage, a positive feedback is created and the alternative pathway can amplify complement activation regardless of the initial activation route. C3 activation products, C3b and iC3b are important opsonins recognized by the phagocyte C3b (CR1) and iC3b (CR3) receptors. After activation of C5, the five terminal plasma glycoproteins (C5b, C6, C7, C8, and C9) bind sequentially to each other to generate the cytolytic membrane attack complex (MAC). Regulation of complement activation occurs at all key steps of the cascade. C1r and C1s are inhibited by the plasma protein C1-inhibitor (C1-INH). C1-INH also inhibits analogous MBL-associated serine protease, MASP-2. Activity of the classical pathway C3/C5 convertase, C4b2a, is inhibited by the plasma factor C4b-binding protein (C4bp). The activity of the alternative pathway C3/C5 convertase, C3bBb, is inhibited by the regulators factor H, DAF, and MCP. On human cell membranes, the main inhibitor of MAC is CD59. Because of the importance of complement as defense and inflammatory mediator system, its deficiencies can predispose to serious diseases. The deficiency in the clearance part (classical pathway) can predispose to SLE, whereas the alternative and terminal pathway deficiencies predispose to microbial infections. Deficiencies of complement regulators predispose to autoreactive disorders, where complement is either excessively activated (HAE, DDD) or misdirected against self cell surfaces (C3GN, aHUS, PNH). Leukocyte adhesion deficiency is a rare consequence of CR3 defect. Abbreviations: SLE, systemic lupus erythematosus; HAE, hereditary angioedema; C1-INH, C1-inhibitor; PNH, paroxysmal nocturnal hemoglobinuria; DDD, dense deposit disease; C3GN, C3 glomerulopathy; aHUS, hemolytic uremic syndrome; LAD, leukocyte adhesion deficiency; DAF, decay-accelerating factor; MASP, mannose-binding lectin-associated serine protease; CR, complement receptor; H, factor H; D, factor D; P, properdin; I, factor I; B, factor B; CD59, protectin; MCP, membrane cofactor protein.

The complement system refers to a group of up to 50 molecules that play a role in various clearance processes and in host defense against microorganisms. Most of these complement components are in blood plasma but some act on cell membranes as receptors for activated components or as regulators that protect host tissues. There are three separate activation routes: the classical, alternative, and lectin pathways. All pathways lead to activation of C3, and continue to the formation of biologically active factors such as C5a, and to the lysis of cells and microbes by the membrane attack complex (MAC). While MAC primarily destroys Gram-negative bacteria, components C1q, C4b, C3b, and iC3b participate in the opsonophagocytic clearance of microbes and endogenous waste products. Activation of the complement system plays an important role in skin defense against microbial infection but also mediates inflammation and tissue injury. A simplified scheme of the complement system is shown in Figure 1.

Skin as a whole is a large organ. It takes part in a spectrum of immunological reactions and is a sensitive indicator of immune dysregulation. Recent studies indicate that skin can have “memory,” i.e., recent immunological reactions can leave a local population of effector or regulatory T cells to activate or downregulate immune reactions, respectively (1). The skin microbiome together with antimicrobial peptides plays a role in immunity. On the other hand, skin reflects several internal injuries of the body and mirrors changes in the immune status of the individual. Keratinocytes of the epidermis of human skin produce several cytokines, chemokines, and many complement proteins. Locally synthesized complement components are believed to play an important role in host defense and inflammation at the organ level. The synthesis of certain complement proteins by human skin keratinocytes and fibroblasts can be either upregulated or downregulated.

Human keratinocytes have been reported to produce the complement inhibitors factor H (FH) and factor H-like protein-1 (FHL-1) as well as the C3b inactivator enzyme factor I for which FH and FHL-1 act as cofactors. The production of these factors is upregulated by interferon-γ (IFN-γ) (2, 3). Recently, Riihilä et al. (4) have presented results indicative of upregulation of FH and FHL-1 in cutaneous squamous cell carcinoma (cSCC) cell lines and in cSCC tumors, also to a lesser extent in cSCC *in situ*. The expression of FH by cSCC cells was upregulated by IFN-γ. Timar et al. (5) have further demonstrated that human keratinocytes produce the terminal complement components C5, C7, C8γ, and C9, but release only C7 and C9. Of the cytokines, tumor necrosis factor alpha (TNF-α) strongly upregulated C9 production. Also C3 (6), factor B (7), complement receptors CR1 and CR2 (8), cC1qR (9), and C5aR (10), and the cell surface complement regulatory proteins membrane cofactor protein (MCP/CD46), decay-accelerating factor (DAF/CD55), and protectin (CD59) (11) have been found to be produced by human keratinocytes. By producing complement factors needed for activation, the skin could augment local complement attack against invading microbes. Most complement components would, however, come from blood plasma, but they get diluted in local areas. Locally produced complement components can compensate for dilution and add extra strength for complement activation, e.g., in areas of local infection. Importantly, not all complement components are needed for opsonophagocytosis of local microbes.

In human skin fibroblasts, TNF-α increases the synthesis of factor B and C3, the activator proteins of the alternative pathway (AP). IL-4, on the contrary, decreases the effect of TNF-α on the synthesis of factor B (12). Synthesis of factor B and C3 are also enhanced by LPS and IFN-γ (13). It has been demontrated that fibroblasts also produce C1q (14) and FH (13), although the main site of the synthesis of FH is the liver. Additionally, complement components C5–C9 have been presented to be produced by fibroblasts (15). Notably, C1q is not produced by the liver (16, 17). Thus, its production is dependent on local synthesis. Macrophages, dendritic cells, and other leukocytes produce C1q. The same holds true for C7, whose synthesis does not take place in the liver (18). Various types of dendritic cells, such as Langerhans cells carry receptors for C1q, C3b, iC3b, and C3d to pick up antigens bound to these factors. Depending on the nature of the antigen, type of opsonin, and phagocytosing cell, the antigens can be processed in different manners.

Decay-accelerating factor regulates autologous complement activation by promoting dissociation of the alternative and classical pathway C3 and C5-convertases on cell surfaces. Peculiarly, DAF is attached to elastic fibers in the extracellular matrix of the dermis (19) and on epidermal cells (20). On cell membranes, DAF expression is usually relatively low compared to other membrane regulators (MCP and CD59), but its level can be readily upregulated. Vitronectin, which prevents the membrane insertion of the MAC, has also been found to associate with elastic fibers in the dermis (21). The significance of these filament or fiber-associated complement regulators is not clear. In some locations, DAF has been shown to act as a receptor for microbes, like for P-fimbriated *E. coli* in the urinary tract. Overall, however, the membrane and soluble inhibitors protect host cells and suppress excessive inflammation, while allowing house-keeping clearance functions and an attack against invading microbes.

In the following text, we will present the most important skin diseases linked to abnormalities in the activity or regulation of the complement system. In addition to the diseases mentioned here, complement plays a role in dermatological inflammatory diseases such as in immediate phototoxic reactions, pustular dermatoses, and in psoriasis as well as in fungal infections (22). Naturally, several other immunopathogenetic mechanisms and signaling events are involved in these diseases, but these pathways will not be discussed here. Because of the inflammatory and cytotoxic effects of complement, it is important and now increasingly possible to control its functions. Therefore, drugs affecting complement activity are also briefly presented.

## Hereditary and Acquired Angioedema

Hereditary angioedema (HAE) is caused by low levels or disturbed functional activity of the complement control protein C1-inhibitor (C1-INH), which is a serine protease inhibitor (serpin) in plasma, also known as SERPING1 (23). C1-INH controls the activities of C1r, C1s, and mannose-binding lectin-associated serine proteases (MASP-1, -2, and -3) of the lectin pathway, factor XII and kallikrein in the contact system, factor XI and thrombin in the coagulation system, and tissue plasminogen activator (tPA) and plasmin in the fibrinolytic system (24). Low levels of C1-INH lead to increased tendency for cleavage of C4 and C2, and patients often have low levels of these proteins. However, levels of C3 are usually not affected.

C1-INH deficiency can be genetic or acquired. Functional C1-INH levels in HAE patients are below 50% of normal. Two main genetic types of C1-INH deficiency are known: in type I HAE (85% of cases), low plasma levels of C1-INH occur, and in type II HAE (15% of cases) C1-INH protein levels are normal but C1-INH is dysfunctional. The latter is due to point mutations in the C1-INH gene. Additionally, a clinical syndrome resembling HAE and termed as type III HAE has been described (25). It affects predominantly women. C1-INH function and its levels are normal. One third of the patients have been found to have a mutation in the clotting factor XII gene. For the rest of the patients, the underlying causes are unknown. One possibility is a defect in proteins that are involved in bradykinin degradation (26–28).

Acquired angioedema (AAE) is characterized by activation of the classical complement pathway and accelerated catabolism of C1-INH. Two different forms of AAE have been described: type I, which is associated with a B cell lymphoproliferative diseases and type II, which is caused by autoantibodies to the C1-INH molecule in otherwise healthy people (29, 30).

**Figure 2:**
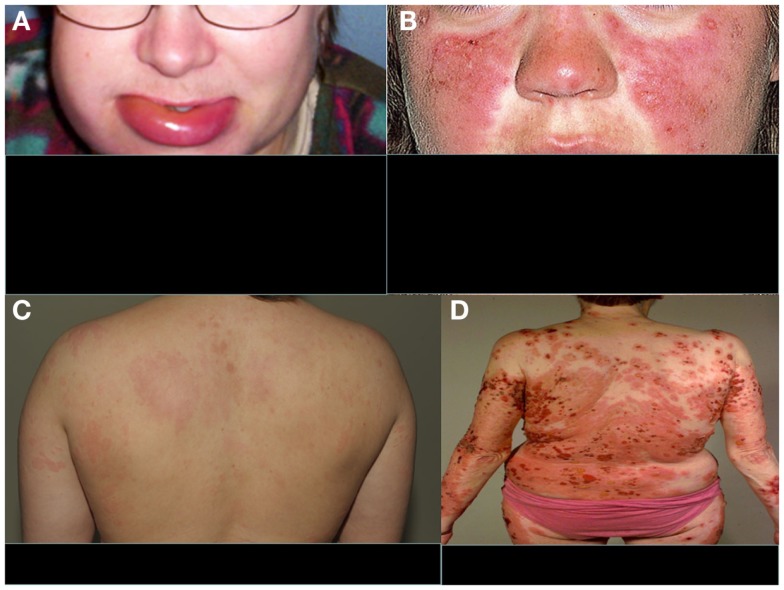
**Typical features of four distinct complement-related diseases with dermatological symptoms**. **(A)** Hereditary angioedema, **(B)** SLE, **(C)** urticarial vasculitis, and **(D)** bullous pemphigoid. **(A,C,D)** are from the photogallery of the Clinic of Dermatology and Allergology, Helsinki University Central Hospital and **(B)** is from http://www.fightinglupus.org/sle-lupus.html

The clinical manifestations of angioedema are due to increased levels of bradykinin as a consequence of uncontrolled cleavage of kininogen by the plasma enzyme kallikrein (28). HAE is characterized by an increased vascular permeability in the deeper layers of the skin and/or the gastrointestinal and laryngeal mucosa representing as angioedema. In addition to mucosal surfaces, attacks of swelling are common at face or at limbs (Figure 2A). The swelling usually lasts 3–4 days. Attacks can be precipitated, e.g., by surgical procedures or stress. They do not respond to therapy by sympatomimetics, antihistamines, or steroids but can be controlled therapeutically by plasma-purified or recombinant C1-INH or by the bradykinin receptor antagonist icatibant. Also, tranexamic acid has been used for the therapy or prevention of HAE attacks. Attenuated androgens, like danazol have been used for prophylaxis, but are nowadays no longer a preferred option.

## Systemic Lupus Erythematosus

Among the strongest, known genetic risk factors for the development of systemic lupus erythematosus (SLE) are deficiencies of the classical complement pathway components (C1q, C1r, C1s, C4, or C2). Of the two C4 isoforms, C4A and C4B, particularly the deficiency of C4A (a “null” allele) is associated with SLE (31). Individuals with complete deficiency of C1q have the highest prevalence of SLE. They usually also develop renal dysfunction as a consequence of the disease (32). Animal studies have indicated that 25% of mice with homozygous C1q deficiency also develop end-stage renal disease (33). It is likely that complement deficiency-related SLE represents one subtype of SLE. The role of the classical pathway in clearing immune complexes has led to the speculation that classical pathway deficiency leads to the development of SLE because of an inability to clear antigen–antibody complexes, chromatin, or other immune aggregates, resulting in their tissue deposition. This process may trigger an inflammatory response and the exposure of autoantigens, with subsequent development of autoimmunity (34). It is possible that, for example, the persistence of chromatin in vascular walls could lead to the development of anti-DNA (and anti-histone) autoantibodies and vasculitis. A common dermatological feature of SLE is the butterfly erythema on the face and photosensitivity (Figure 2B).

Systemic lupus erythematosus or SLE-like disease can be caused by multiple mechanisms. Often, SLE is related to excessive apoptosis and production of cellular waste or its defective clearance. A common thought is that apoptotic cells represent a challenge to immune tolerance. It has been demonstrated that apoptotic keratinocytes (rendered apoptotic by ultraviolet B radiation or viral infection) are specifically recognized by human C1q in an antibody-independent manner (32). Furthermore, there is evidence that viral and self-antigens are concentrated and clustered in blebs of apoptotic keratinocytes (35). This has led to the suggestion that C1q may be critical for proper recognition, clearance, and processing of self-antigens contained within apoptotic surface blebs. About 20–40% of SLE patients have autoantibodies to the collagen-like region (CLR) of C1q (36), but many SLE patients also have autoantibodies directed to the globular domain of C1q (37). In some SLE patients, a low molecular weight form of C1q is produced (38) resulting in functional C1q deficiency.

Levels of anti-C1-INH IgG antibodies have been shown to be significantly higher in SLE patients than in controls, and to correlate with the duration and activity of the disease. However, no correlation between anti-C1-INH and anti-C1q IgG antibodies was found (39). It has also been reported that levels of antibodies directed against mannose-binding lectin (MBL) are significantly higher in patients with SLE compared to healthy subjects. No significant difference in the anti-MBL antibodies was found between patients with active or inactive disease (40). Epidemiological studies on SLE cohorts have not indicated MBL deficiency as a susceptibility factor for SLE (41).

## Vasculitic Syndromes and Urticarial Vasculitis

Circulating C3dg and soluble terminal complement complexes (SC5b-9) as well as perivascular deposits of C3d and MAC have been found in skin lesions of patients with vasculitis (42). These are all evidence for both systemic and local complement activation. MAC has been detected in the vascular walls of skin samples from patients with Henoch–Schönlein purpura and SLE (43, 44). In different forms of vasculitis, including leucocytoclastic vasculitis, C3 with IgG is usually seen on endothelial cells of the dermal vessels by immunofluorescence (IF) microscopy. The kallikrein–kinin system has been reported to be activated in vasculitis leading to the release of bradykinin. Complement activation on the endothelium leads to MAC formation, cell activation with consequent production of chemokines, membrane blebbing, and release of membrane microparticles. C3a and C5a induce endothelial cytokine release and an increase in vascular leakage. The terminal complement complex C5b-9, either in a soluble form (SC5b-9) or as MAC, can induce increased endothelial permeability (45, 46). Recent findings suggest that stimulation of neutrophils by anti-neutrophil cytoplasmic antibody (ANCA) causes release of factors that activate complement via the alternative pathway, thus initiating an inflammatory amplification loop that mediates the severe necrotizing inflammation of ANCA-associated vasculitis (47). Thus, both the complement and the kinin systems are thought to become activated during vasculitis and play a central role in disease pathogenesis.

Hypocomplementemic urticarial vasculitis syndrome (HUVS) is associated with anti-C1q antibodies and low levels of the classical pathway components, namely C1q, C2, C4, and C3. The possible mechanisms of vascular damage include formation of immune complexes, anti-C1q antibodies, and a T-lymphocyte response (48). In addition, anti-C1q antibodies may interfere with the clearance of apoptotic cells, influencing induction and expression of autoimmunity (49). HUVS presents as a clinically more aggressive and long-standing disease (>6 months) with urticarial burning and pruritic lesions than normocomplementemic urticarial vasculitis (Figure 2C). Additionally, clinical findings such as leucocytoclastic vasculitis, severe angioedema, pulmonary involvement, arthritis, glomerulonephritis, and recurrent abdominal pain are possible. Anti-C1q antibodies have been described also, e.g., in SLE and Sjögren’s syndrome, and some patients with HUVS may have clinical features of these connective tissue diseases (50).

## Bullous Skin Diseases

### Pemphigus

Pemphigus, a group of bullous diseases affecting skin and oral mucosa, is caused by an antibody-mediated autoimmune reaction to desmogleins (Dsg), desmosomal transmembrane glycoproteins. Autoantibodies have been suggested to activate the complement system leading to loss of cohesion of individual epidermal keratinocytes (acantholysis) and intraepidermal blisters. Pemphigus is classified into pemphigus vulgaris (PV), with suprabasal acantholysis, and into pemphigus foliaceus (PF) and pemphigus erythematosus (PE), with acantholysis in the more superficial epidermis. PV is characterized by IgG autoantibodies against Dsg 3, while the target in PF and PE is Dsg 1. About 50% of patients with PV have also Dsg 1 autoantibodies. PV patients with oral mucosal lesions have predominantly Dsg 3 antibodies, while PF patients with Dsg 1 do not have mucosal symptoms (51, 52). Dsg antigens are localized in the intercellular substance (ICS) of the epidermis and autoantibodies to ICS are also present in patients with pemphigus. Components of both the classical and the alternative pathway including C1q, C4, C3, and properdin (P) are found in the ICS in PV lesions (53). It is assumed that a direct toxic effect of MAC on epidermal cells plays a role in the pathogenesis because, in addition to C1q, C3, and C4, also C5, C7, and C9 and the MAC-neoantigen have been found in the ICS area both in PV and PF (54). Complement consumption and activation products can be observed in the blister fluids. In comparison, complement levels in the blister fluids produced on the skin of normal individuals are equivalent to those in serum, and the levels of activation products are very low. This indicates a disease-specific activity. IF microscopy analysis of the skin biopsies shows usually C3 and IgG between keratinocytes. Tissue-bound and circulating IgG autoantibodies in pemphigus patients mainly belong to IgG1 and IgG4 subclasses, of which IgG1 is a potent complement activator (55).

### Bullous pemphigoid

Bullous pemphigoid (BP) is a generalized blistering disorder characterized by pruritus and rigid, subepidermal blisters (Figure 2D). Autoantibodies against the main BP antigen BP180 and its NC16A domain (located in collagen XVII) are seen in this disease. The target antigen is localized to hemidesmosome, the main epidermal structure maintaining adherence of the epidermis to the basement membrane (56, 57). Subepidermal blistering is initiated by anti-BP180 antibodies binding to the basement membrane zone (BMZ) and mediated by complement activation via the classical pathway, mast cell degranulation, and neutrophil infiltration (58, 59). Complement activation and chemotactic peptides attract neutrophils and other inflammatory cells to the site. Complement components present in the region of blister formation include C1, C3, C3d, P, C5, and MAC. Direct IF staining of the skin biopsy shows usually C3 and IgG at BMZ.

A disease belonging to the pemphigoid group is linear IgA-dermatosis, also called the chronic bullous disease of childhood. Here, the antigen is also BP180/NC16A but direct IF of the skin biopsy shows linear IgA fluorescence. Also IgA antibodies against the NC16A domain of BP180 have been found (60). IgA does, however, not activate the complement system. On mucosal membranes, IgA competes with IgG and thereby can prevent complement activation. Nevertheless, aggregates of heavily glycosylated or modified IgA, like in IgA nephropathy, could activate the alternative pathway of the complement system and contribute to disease pathogenesis.

### Mucous membrane pemphigoid

Mucous membrane pemphigoid, also called as cicatricial pemphigoid (CP) is a heterogeneous disease with subepidermal blistering. The main antigen in this disease is also BP180 protein but various other autoantigens have been identified including laminin 5 (epiligrin) and α6β4 integrin. Autoantibodies against BP180 are thus often negative in this patient group and the diagnosis is mainly based on direct IF of the mucosal biopsy, which shows deposition of IgG, C3, and IgA at the BMZ (59). Autoantibodies in CP mainly belong to IgG4 and IgG1 subclasses, while anti-epiligrin CP autoantibodies against laminin 5 belong usually to the IgG4 subclass (55). Because IgG4 is not a complement-activating IgG subclass, sera from patients with autoantibodies against laminin 5 do not fix C3 to the epidermal basement membrane and do not induce leukocyte-dependent dermal–epidermal separation *in vitro* (55). This suggests that complement activation does not play a major role in all forms of CP.

### Epidermolysis bullosa acquisita

A similar kind of complement activation with dermoepidermal separation as in BP is seen also in epidermolysis bullosa acquisita (EBA) and in pemphigoides gestationis. Complement-induced inflammation is pronounced in EBA, where subepidermal blisters develop at the sites of trauma leading to atrophic scars (22). Circulating and tissue-bound autoantibodies directed against type VII collagen are characteristic for EBA. Type-VII collagen forms anchoring fibrils that connect the epidermis and the BMZ to the papillary dermis. Autoantibodies against type-VII collagen are able to activate the complement system *in vitro* and *in vivo*. Deposition of various complement components, including C3b and MAC, are commonly found in the skin of EBA patients (59).

### Pemphigoides gestationis

In pemphigoides gestationis (pemphigoid of pregnancy, earlier referred to as herpes gestationis) subepidermal blisters arise during the second and third trimesters of pregnancy. Autoantibodies against BP180 antigen are found also in this disease. Linear deposition of C3b and, to a lesser extent, of IgG is seen at BMZ by direct IF microscopy.

### Dermatitis herpetiformis

Dermatitis herpetiformis (DH), a celiac disease-related skin disorder, is characterized with itchy blistering skin lesions especially at elbows and knees. The major antigen, as in the celiac disease, is tissue (=type 2) transglutaminase (tTG) although epidermal (=type 3) transglutaminase (eTG) has been identified within the papillary IgA granules in BMZ in DH. Direct IF microscopy shows mainly IgA but also C3 and IgM in the small vessels of papillary dermis. A subpapillary vascular fluorescence is also sometimes observed. Skin IgA is colocalized with eTG in the vessel walls, which indicates a role for anti-eTG antibodies in the pathogenesis of skin symptoms (61). As mentioned before, IgA does not usually activate the complement system.

## Partial Lipodystrophy

Partial lipodystrophy (PLD) is a rare disease characterized by a symmetric loss of subcutaneous fat usually from the face, arms, and trunk. It is associated with an abnormal complement activator, the C3 nephritic factor (C3NeF), low C3 levels, and membranoproliferative glomerulonephritis type II (MPGN II) (62). Because of dense intramembranous deposits of C3b and components of MAC in glomerular basement membranes, MPGN II is nowadays called as dense deposit disease or DDD (63). C3NeF is an IgG autoantibody, which binds to and stabilizes the alternative pathway C3 convertase (C3bBb). It promotes C3 activation, causing C3 consumption. The onset of the disease is usually in early childhood, sometimes following an acute viral infection. The mechanism of lipodystrophy is unknown, but could be related to small vessel vasculitis caused by C3b deposition. Adipose cells produce substantial amounts of factor D (also known as adipsin). Thus, it is also possible that hyperactivation of the alternative pathway in the vicinity of or targeted at fat cells could cause direct damage to them.

Partial lipodystrophy has been reported to be associated with autoimmune disorders such as Sjögren’s syndrome, myasthenia gravis, idiopathic thrombocytopenic purpura, Raynaud’s phenomenon, scleroderma, Hashimoto’s disease, and SLE (62, 64). An additional autoantibody, C4 nephritic factor (C4NeF), has been found to coexist in some patients with MPGN II and hypocomplementemia (65). In additon, Savage et al. (66) have reported three patients with acquired generalized lipodystrophy, low C4 levels, and autoimmune hepatitis. The complement system is linked to lipid metabolism by multiple ways and MAC can destroy adipocytes, but why complement hypercatabolism causes PLD is not fully understood.

## Complement Deficiency States and Skin Infections

Pyogenic skin infections have been associated with the lack of C2 as well as with factor I deficiency. Factor I is a fluid-phase inactivator of C3b and C4b thus influencing both the alternative and classical pathways. The deficiency leads secondarily to C3 and factor B deficiencies. Also C8 deficiency has been associated with chronic pyogenic infections or with xeroderma pigmentosa (67), but apparently this is a rare association. The lack of C4A is associated with increased likelihood of recurrent severe herpes infections (68). Severe meningococcal infections are often linked to immunodeficiencies. Risk of invasive neisserial infections is increased especially in deficiencies of properdin or of the terminal complement components (C5, C6, C7, C8, or C9) (69, 70).

The complement receptors CR1 and CR3 are expressed predominantly on leukocytes (CR1 also on erythrocytes and glomerular podocytes) and CR2 on B cells and dendritic cells. Rare patients with a complete deficiency in CR3 have repeated infections particularly on the skin and mucosal membranes, which heal leaving paper-thin scars. It is thought that CR3 deficiency leads to leukocyte adhesion deficiency (LAD) and an inability of the CR3-deficient neutrophils to mount a respiratory burst in response to a phagocytic stimulus (67). The integrin β-chain (CD18) and subsequently the CD11a/18, CD11b/18 (CR3), and CD11c/18 (CR4) receptors are absent in LAD.

## Skin Manifestations Caused by Microbes Evading Complement Attack

### Borreliae

Lyme borreliosis (LB) is caused by spirochetes belonging to the *Borrelia burgdorferi* sensu lato group. The main causative subspecies are *B. afzelii*, *B. garinii*, and *B. burgdorferi* sensu stricto, although new subspecies *B. valaisiana*, *B. spielmanii* (71), *B. lusitaniae*, and *B. bissettii* (72) have also been reported in Europe. Cutaneous symptoms include early-stage erythema migrans, lymphadenosis benigna cutis (lymphocytoma), and late-stage acrodermatitis chronica atrophicans (ACA) (73). Lymphocytoma and ACA are predominantly caused by *B. afzelii* and therefore mostly seen only in Europe. In the US and Canada, *B. burgdorferi* sensu stricto is the main causative agent of LB: arthritis often preceeded by erythema migrans.

**Figure 3:**
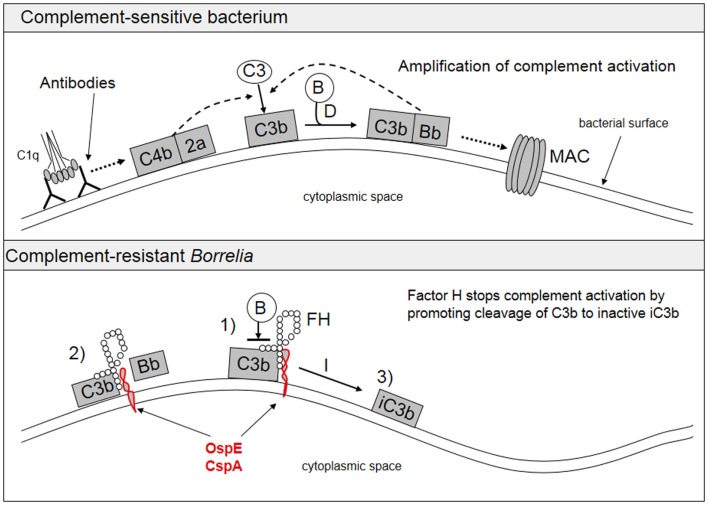
**Activation and inhibition of the complement system on complement sensitive and resistant bacteria**. The upper part shows the normal situation in complement activation, where a microbe becomes a target for complement attack. The lower part shows how borrelial OspE/CspA (or any other similar microbial protein) binds factor H to block complement activation on the bacterial surface. FH, factor H; B, factor B.

A typical feature of *Borrelia* spirochetes is their ability to escape immune clearance and survive for a long time in their human or animal hosts. To protect themselves and to avoid attack by the complement system, *Borrelia* spirochetes bind soluble complement regulatory proteins to their surfaces. There is evidence that serum-resistant *B. burgdorferi* sensu stricto and *B. afzelii* are able to bind complement inhibitors FH and factor H-like protein-1 (FHL-1) (74–76) as well as the classical pathway inhibitor C4bp (77). We have also demonstrated that all borrelial subspecies causing human infections, including several strains of *B. garinii*, carry *ospE* genes to protect themselves against complement attack *in vivo* (78). FH and FHL-1 control C3b formation by inhibiting the formation and activity of the AP C3 convertase enzyme, C3bBb, and by acting as cofactors for factor I-mediated inactivation of C3b. The binding of FH to the borrelial surface occurs via two main proteins, outer surface protein E (OspE) and CspA, also called complement regulator-acquiring surface protein-1 (CRASP-1) (74–76, 79, 80). Several plasmid-encoded OspE paralogs have been reported to be responsible for FH binding to *Borrelia* and to mediate complement resistance (81, 82). We have previously presented that OspE interacts with the C-terminal short consensus repeats (SCRs) 19–20 of FH (75, 83). Figure 3 shows a scheme of complement activation and inhibition on complement sensitive and resistant bacteria.

### Group A *Streptococcus*

Group A *Streptococcus* (*Streptococcus pyogenes*) is one of the most important human pathogens. It causes skin diseases such as impetigo (pyogenic skin infections), erysipelas, scarlet fever, cellulitis, and wound infections. It also acts as a trigger for skin purpura and vasculitis or for psoriasis guttata. A more severe septic form of a skin manifestation is necrotizing fasciitis, and *S. pyogenes* can also cause streptococcal toxic shock syndrome.

The major surface protein of *S. pyogenes* is M protein, which is involved in the protection of the bacterium from phagocytosis. The main immunological systems for elimination of the bacteria are the complement system, antibodies, and phagocytic cells. Because of a possible capsule and thick peptidoglycan layer on the bacteria, MAC is not effective in directly killing of the bacteria. Several specific ways of *S. pyogenes* to avoid complement attack have been described. These include acquisition of host inhibitors of C3- and C5-convertases (FH and C4bp), a C5a chemotaxin cleaving enzyme (C5a peptidase) and a suggested inhibitor of the terminal complement complexes (streptococcal inhibitor of complement, *SIC*). By binding antibodies via the Fc-region, the bacteria can also inhibit immunoglobulin-mediated complement activation and opsonization (84–87). As the binding of complement FH is important in the pathogenesis of *S. pyogenes* infections, its inhibition could provide a novel therapeutic approach (88). A variant of FH (Y4024) that predisposes to age-related macular degeneration protects against severe group A streptococcal infections, because the bacteria cannot bind this variant so well to their surfaces (88).

### *Staphylococcus* *aureus*

*Staphylococcus aureus* is a major human pathogen causing diseases ranging from superficial skin infections and abscesses to invasive infections such as osteomyelitis, endocarditis, and sepsis (89). This pathogen can secrete small proteins that interfere with functions of the complement component C3 and suppress complement activation leading to inhibition of opsonization and blocking generation of chemotactic C3a and C5a. A key inhibitor is SCIN, the staphylococcal inhibitor of the C3bBb convertase. *S. aureus* can also enhance FH-mediated complement regulation on the microbial surface. This occurs by secreting the extracellular protein Ecb, which promotes binding of FH to C3b to downregulate complement activation (90). *S. aureus* can thereby use host FH and bacterial Ecb in a concerted action to eliminate C3b at the site of infection. *S. aureus* binder of IgG (Sbi) can prevent binding of IgG to Fcγ receptors and modulate binding of C3b to FH (91). The N-terminus of the extracellular fibrinogen binding protein (Efb) inhibits platelet function by binding fibrinogen and its C-terminal part binds to the C3d-domain of C3 and C3b (92, 93). Both Sbi and Efb are also suggested to be able to form tripartite complexes with host complement FH (91, 94). A recent study indicated that Efb can block neutrophil-mediated phagocytosis of *S. aureus* (95).

## Therapeutic Possibilities for Complement-Mediated Diseases

### C1 esterase inhibitor

C1 esterase inhibitor concentrate from human plasma (Berinert^R^, Cetor/Cebitor^R^, Cinryze^R^) has been in clinical use for treating acute type I and II HAE since 1973. In the beginning, it was regarded as an emergency therapy only. After the availability of purified and virus-inactivated, C1-INH concentrates the indication was extended stepwise to milder forms of the disease (96). C1-INH concentrates at doses of 20 U/kg provide a fast relief of symptoms for HAE attacks (97). The treatment effects are similar in facial and abdominal attacks, as well as in moderate and severe attacks. Additionally, a recombinant analog of C1-INH concentrate, alphaconestate (Ruconest^R^), has become available for clinical use in type I and type II HAE. In the US, a recombinant protein kallikrein inhibitor ecallantide (Kalbitor^R^) has been approved for the treatment of HAE. C1-INH concentrate has also been used with improvement in prognosis for patients with capillary leakage syndrome following bone marrow transplantation (98).

### Icatibant

Icatibant (Firazyr^R^) is a bradykinin type 2 (B2) receptor antagonist. It is a synthetic decapeptide used for treating the autosomal dominant type of HAE lacking C1-INH. It controls the effects of increased levels of bradykinin seen in HAE. Thus, it does not directly influence the complement levels. As an example of an off-label indication, icatibant was used successfully in severe capillary leakage syndrome caused by a Puumala hantavirus infection (99).

### Eculizumab

Eculizumab (Soliris^R^) is a long-acting humanized monoclonal antibody against complement C5. It inhibits the cleavage of C5 into C5a and C5b hence blocking the generation of C5a anaphylatoxin and formation of MAC. This treatment was initially registered for patients with paroxysmal nocturnal hemoglobinuria (PNH), where an acquired defect in the glycophosphoinositol-anchored natural complement inhibitors, CD55 and CD59 leads to complement-mediated hemolysis (100). Recently, eculizumab has been registered also for the use in atypical hemolytic uremic syndrome (aHUS) and it has been used also for the treatment of thrombotic thrombocytopenic purpura (101). Since eculizumab practically converts the patients deficient in the terminal C pathway, their susceptibility to meningococcal disease increases. Therefore, appropriate prophylaxis, vaccination against *Neisseria meningitidis* and antibiotics, is required before the therapy can be started.

### Microbial complement inhibitors as vaccines

Complement inhibiting surface proteins of pathogenic bacteria have been suggested as candidates for future, function-based, vaccines (102). An immune response against such surface protein would recognize the microbe and neutralize its complement resistance, which is key bacterial virulence mechanism. Bacterial complement inhibitors often bind the soluble inhibitors FH or C4bp. They would act as vaccine candidates for preventing diseases such as group B meningococcal meningitis, systemic pneumococcal or group B streptococcal disease, and LB. In fact, serogroup B meningococcal factor H binding protein (FHBP) is already a component in a recently approved new vaccine (Bexsero^®^, Novartis).

## Conclusion

The complement system plays both protective and harmful roles in disease. Better understanding of the complex interactions between various components of the complement pathway and their interactions with other immune pathways as well as with the coagulation and kinin systems are essential for understanding various aspects of disease pathogenesis. Characterization of the role of the complement system in the diseases may lead to better therapeutic opportunities. The availability of effective C inhibitors for the classical pathway (C1-INH) and for the terminal pathway (eculizumab, anti-C5) provides new opportunities to treat life-threatening diseases like HAE, PNH, and aHUS.

## Conflict of Interest Statement

The authors declare that the research related to this article and writing was conducted in the absence of any commercial or financial relationships that could be construed as a potential conflict of interest.
